# Response to Letter to the Editor: Can Quantitative Pupillometry be Used to Screen for Elevated Intracranial Pressure? A Retrospective Cohort Study

**DOI:** 10.1007/s12028-022-01550-y

**Published:** 2022-06-27

**Authors:** Jakob Pansell, Peter Rudberg, Max Bell, Charith Cooray

**Affiliations:** 1 Department of Clinical Neuroscience, Karolinska Institutet, Stockholm, Sweden; 2 Department of Anesthesia and Intensive Care Medicine, Karolinska University Hospital, Karolinska Institutet, Stockholm, Sweden; 3 Department of Physiology and Pharmacology, Karolinska Institutet, Stockholm, Sweden; 4 Department of Clinical Neurophysiology, Karolinska University Hospital, Karolinska Institutet, Stockholm, Sweden

We would like to thank Professor Maas and colleagues [1] for showing interest in our recent publication, “Can Quantitative Pupillometry be Used to Screen for Elevated Intracranial Pressure? A Retrospective Cohort Study”. We welcome this discussion regarding the use of quantitative pupillometry. The arguments and reasoning regarding how to interpret negative predictive value (NPV) and positive predictive value (PPV) outlined by Maas et al. [1] in their letter to the editor are completely valid. However, we believe that Maas and colleagues [1] have interpreted our conclusion somewhat differently than what we intended. We stated as our main conclusion in the abstract, “Screening with NPi may inform high stakes clinical decisions by *ruling out* elevated ICP with a high degree of certainty” (emphasis added) [2].

Our high NPV of 96.7% supports this conclusion, suggesting a very low rate of false negative results.

NPV is, as Maas et al. [1] outline, dependent on the prevalence of the condition.

Our results are based on a population undergoing invasive intracranial pressure (ICP) monitoring, and although the proportion of elevated ICP in our cohort was “merely” 7%, it is difficult imagining a screening population with a higher rate of elevated ICP. Rather, the prevalence of elevated ICP in a broader population with a mere suggestion of elevated ICP would be even lower, which would be reflected as an even higher NPV. As Maas et al. [1] correctly state, PPV would worsen with a lower pretest probability, but this is not the main purpose of implementing a rule-out test. Compare this with the widely used D-dimer test for ruling out venous thromboembolism; this test has an excellent NPV but a poorer PPV, yet it still finds important clinical use as a rule-out test [3].

Clinical decisions always involve weighing different risks and opportunities for the individual patient. In some decisions, high sensitivity and PPV are preferable, whereas in other decisions high specificity and NPV are preferable.

We agree with Maas et al. [1] that quantitative pupillometry in the hospital setting most likely has its main use as part of a multimodal approach to neuromonitoring, and we currently have ongoing studies of that, as well. However, given the ease of use and excellent interrater reliability of quantitative pupillometry [4–6], we believe that it may be of use in low-resource and/or prehospital settings, as well, as a *rule-out test*. The use of quantitative pupillometry is largely to be decided by future research. Still, based on current knowledge, we hold to the cautious statement in our conclusion that quantitative pupillometry may inform high-stake clinical decisions by ruling out elevated ICP with a high degree of certainty, especially under circumstances in which clinicians have little information—except for clinical findings—to base their decisions on.
